# Development and Temporal Validation of a Prediction Model for PICC‐Related Catheter‐Associated Skin Injury

**DOI:** 10.1002/nop2.70895

**Published:** 2026-10-09

**Authors:** Xianghong Jin, Xiuzhu Cao, Yidan Chen, Linfang Zhao

**Affiliations:** ^1^ Nursing Department Sir Run Run Shaw Hospital, Zhejiang University School of Medicine Zhejiang Hangzhou China

**Keywords:** catheter related skin injury, peripherally inserted central catheter, prediction model

## Abstract

**Objective:**

To identify predictors of peripherally inserted central catheter (PICC)‐related catheter‐associated skin injury (CASI) and to develop and temporally validate a bedside prediction model.

**Methods:**

In this prospective single‐centre cohort study, 1309 adults who underwent PICC insertion from June 2022 to June 2023 formed the development cohort and 568 adults enrolled from July to September 2023 formed the temporally separated validation cohort. Participants underwent prospective weekly skin assessment until PICC removal or 6 months. Candidate predictors were selected using LASSO regression with 10‐fold cross‐validation and the one‐standard‐error rule, followed by multivariable logistic regression. The final six‐predictor model was assessed using bootstrap internal validation, temporal validation, calibration and decision‐curve analysis.

**Results:**

PICC‐related CASI occurred in 116 of 1309 participants (8.86%) in the development cohort and 40 of 568 (7.04%) in the validation cohort. The final model included primary tumour diagnosis (OR 3.86, 95% CI 1.30–11.50), history of catheterization (OR 5.08, 95% CI 2.67–9.67), iodophor disinfectant use (OR 0.11, 95% CI 0.05–0.25), functional dressing use (OR 0.16, 95% CI 0.06–0.41), cytotoxic drug use (OR 4.03, 95% CI 1.70–9.55) and hormonal drug use (OR 2.17, 95% CI 1.09–4.35). The optimism‐corrected AUC was 0.811 in the development cohort. In temporal validation, the AUC was 0.681 (95% CI 0.610–0.743).

**Conclusion:**

The six‐predictor model demonstrated good discrimination in the development cohort and may assist clinicians in identifying patients at high risk of PICC‐related CASI. However, as a development‐stage prediction model, it requires recalibration and independent multicentre external validation before being considered for routine clinical use.

**Implications for Nursing Practice:**

The identified predictors may help nurses recognize patients at higher risk of PICC‐related CASI and support more structured skin assessment during PICC care. As the model remains at the development stage, further recalibration and multicentre external validation are needed before routine clinical use.

Peripherally inserted central catheters (PICCs) are widely used for medium‐ to long‐term vascular access in patients receiving anticancer treatment, intensive therapy, parenteral nutrition and other prolonged intravenous therapies. PICC‐related catheter‐associated skin injury (CASI) is defined as an abnormality, including but not limited to erythema, vesicle, bulla, erosion or tear, observed in the area of the dressing or securement device for 30 min or longer after its removal (Nickel et al. 2024). Reported rates vary by population and definition; Ullman et al. reported 22.5 skin complications per 1000 catheter‐days for central vascular access devices (Ullman et al. 2019) and Wang et al. reported an incidence of 40.8% in patients with chest tumours (Wang et al. 2023). CASI may impair comfort, interrupt treatment, contribute to unplanned catheter removal and increase the risk of further catheter‐related complications.

Early identification of high‐risk patients is essential for the prevention of PICC‐related CASI (Broadhurst et al. 2017). Previous studies have included a risk assessment scale for medical adhesive‐related skin injury at PICC insertion sites in patients with cancer (Li, Zhang, et al. 2023), a predictive nomogram for medical adhesive‐related skin injury among patients with central venous access devices (Li, Hao, et al. 2023) and observational studies investigating skin complications associated with PICCs or central venous access devices (Wang et al. 2023; Gavin et al. 2024). However, these tools and studies have not fully addressed the broader construct of CASI as defined by the INS/WoCoVA framework and therefore provide only partial guidance for the prediction of PICC‐related CASI (Li, Zhang, et al. 2023; Li, Hao, et al. 2023).

Accordingly, the present study focused on this specific and clinically relevant evidence gap. We aimed to identify predictors associated with PICC‐related CASI and to develop a risk prediction model for identifying high‐risk patients (Balachandran et al. 2015), thereby providing a reference for clinical risk stratification and the development of targeted preventive strategies.

## 1 Study Subjects and Methods

### 1.1 Study Subjects

This prospective cohort study used convenience sampling to enrol adults who underwent PICC placement at a single comprehensive tertiary hospital in China between June 2022 and September 2023. The intended prediction time was PICC insertion, after the initial catheter‐maintenance plan had been formulated. Outcome ascertainment was prospective: trained investigators assessed the insertion‐site skin at weekly follow‐up visits until PICC removal or 6 months, whichever occurred first. All PICCs were inserted and maintained within a well‐established specialist intravenous therapy service. Inclusion criteria: Age ≥ 18 years; Patients who underwent PICC placement at this hospital; Expected catheter dwell time > 7 days; Patients who were expected to receive all catheter maintenance at this hospital and to complete follow‐up for up to 6 months. Exclusion criteria: Patients with pre‐existing skin lesions, such as rashes, damage, oedema or scars at the puncture site at the time of catheter insertion; Patients with concurrent skin diseases; Patients requiring emergency catheter insertion in resuscitation situations.

This study was approved by the Ethics Committee of Sir Run Run Shaw Hospital, Zhejiang University School of Medicine, approval No. [2022] (0307) and all procedures were conducted in accordance with the principles of the Declaration of Helsinki.

### 1.2 Sample Size Calculation

This study was designed as a prospective cohort study for the development and temporal validation of a prediction model. No formal sample‐size calculation based on the pmsampsize framework was performed before participant recruitment (Riley et al. 2020). All clinically relevant candidate predictors were prespecified according to previous literature and expert opinion and were entered directly into the LASSO logistic regression model without univariable prescreening. Therefore, despite LASSO selection and bootstrap internal validation, model overfitting cannot be excluded.

A total of 1877 participants met the eligibility criteria, including 1309 in the development cohort and 568 in the temporal validation cohort. The development cohort contained 116 CASI events and the validation cohort contained 40 events.

### 1.3 Research Methods

#### 1.3.1 Data Collection Tools

Based on a review of the literature and expert consultation, the research team developed a standardized data collection form for PICC‐related CASI. The form covered six domains of candidate predictors: demographic characteristics (e.g., sex, age, height, weight, smoking and alcohol use), disease‐related information (e.g., primary diagnosis, allergy history, hypertension, diabetes and kidney disease), nutritional status (e.g., BMI, serum albumin and haemoglobin), treatment‐related information (e.g., cytotoxic, targeted, immunotherapy and hormonal therapies), catheter‐insertion characteristics (e.g., insertion site, catheter material and catheter diameter) and catheter‐maintenance practices (e.g., dressing, disinfectant and fixation products). PICC‐related CASI outcome information was collected separately (whether it occurred, symptom description, Type classification). In total, 38 prespecified candidate predictors, excluding the outcome, were included in the model‐development analysis. The complete list of candidate predictors, coding and assessment timing is provided in Table S1.

#### 1.3.2 Data Collection Methods

Data collection began after written informed consent was obtained and participants were enrolled. The first study visit was performed on the day of PICC insertion. Baseline demographic characteristics, disease‐related information, laboratory test results and catheter‐insertion information were obtained from the hospital information system and the vascular‐access team at that visit. The routine antiseptic and dressing strategy was determined at PICC insertion or when the initial maintenance plan was formulated. Accordingly, iodophor disinfectant use and functional dressing use were defined from the strategy known at the intended prediction time; subsequent changes made after CASI or another complication did not redefine these baseline predictors. Cytotoxic and hormonal therapy were defined from information already available at the prediction time: treatment was either already ongoing or had been included in the established treatment plan at PICC insertion. Actual administration could occur later and was prospectively confirmed during follow‐up, but treatment newly initiated after PICC insertion that had not been part of the baseline plan was not used to redefine these predictors.

Participants were subsequently followed up weekly until the first occurrence of PICC‐related CASI, PICC removal or the 6‐month study endpoint. During follow‐up, insertion‐site skin condition, medication administration, catheter maintenance and nutritional status were prospectively recorded for outcome ascertainment and clinical follow‐up. For prediction modelling, BMI, serum albumin and haemoglobin were defined using baseline values available at the first study visit, before or at PICC insertion and before the intended prediction time. BMI was calculated from baseline height and body weight and serum albumin and haemoglobin were classified from the corresponding baseline laboratory results. There were no missing data for the prespecified candidate predictors or the outcome in the development and validation datasets; therefore, no imputation was required and a complete‐case analysis was performed.

PICC‐related CASI was defined according to the INS Infusion Therapy Standards of Practice (Standard 52) (Nickel et al. 2024). Clinical classification was based on the algorithm proposed by Broadhurst et al. (Broadhurst et al. 2017), including irritant/allergic contact dermatitis, exit‐site infection, skin injury (including skin stripping, skin tears and tension blisters) and non‐infectious exudate or leakage. All suspected CASI lesions were photographed, coded and archived. Cases with uncertain classification were jointly reviewed by the principal investigator and a dermatologist to reach a final diagnosis.

### 1.4 Statistical Methods

Statistical analyses were performed using SPSS version 26.0 and R version 4.4.0. Continuous variables with a normal distribution are presented as mean ± standard deviation (SD). Non‐normally distributed continuous variables are presented as median (interquartile range, IQR). Categorical variables are expressed as frequencies and percentages.

Patients were classified into the CASI group and the non‐CASI group according to the occurrence of PICC‐related CASI. Patients who underwent PICC insertion between June 2022 and June 2023 were included in the development cohort, whereas those who underwent PICC insertion between July 2023 and September 2023 were included in the temporal validation cohort. Baseline characteristics of participants with and without CASI were summarized descriptively for clinical interpretation only and were not used for predictor selection. All 38 prespecified candidate predictors were entered directly into the LASSO logistic regression model without univariable prescreening. Candidate predictors were selected using LASSO logistic regression with 10‐fold cross‐validation and the penalty parameter was chosen using the one‐standard‐error criterion (lambda.1se). The selected predictors were subsequently entered into an unpenalized multivariable logistic regression model. Bootstrap internal validation used 1000 resamples of the final fitted model; the LASSO selection step was not repeated within each bootstrap resample, so the bootstrap correction does not fully incorporate uncertainty from variable selection. Model performance was evaluated using AUC, calibration curves, temporal validation and decision‐curve analysis. All statistical tests were two‐sided and *p* < 0.05 was considered statistically significant. Reporting of this prediction‐model study followed the TRIPOD + AI guidance; the completed checklist is provided in Table S2.

### 1.5 Quality Control

All investigators received standardized training in eligibility assessment, follow‐up and CASI classification. Data were entered in duplicate and checked against the source records. Most baseline variables were obtained from the electronic hospital platform, whereas weekly outcome assessment and selected history variables were collected prospectively by the research team.

## 2 Results

### 2.1 General Information of Patients

A total of 1973 patients were assessed for eligibility, of whom 1877 were included in the final analysis. The development cohort comprised 1309 participants and the temporal validation cohort comprised 568 participants. Among them, 78 cases were withdrawn because they were transferred back to local hospitals and could not complete the weekly skin assessments and 18 cases were withdrawn due to patient refusal to return for follow‐up visits.

The incidence of CASI was 8.31% (156/1877). Among the 1309 patients in the development cohort, there were 768 males (58.70%) and 541 females (41.30%); the average age was (62.04 ± 13.76) years. This group included 878 patients with tumours from various systems such as the respiratory, digestive, reproductive, urinary, circulatory, immune, neuroendocrine and musculoskeletal systems, as well as 431 patients with other non‐tumour diseases. The incidence of CASI was 8.86% (116/1309). In the temporal validation cohort of 568 patients, there were 332 males (58.50%) and 236 females (41.50%); the average age was (62.08 ± 12.87) years, including 410 patients with tumours and 158 patients with other non‐tumour diseases. The incidence of CASI in this group was 7.04% (40/568).

### 2.2 Current Status of PICC‐Related CASI

Among the 1309 patients in the development cohort, the incidence of PICC‐related CASI was 8.86% (116 cases). Of these, 81.90% (95 cases) were classified as mild, 9.48% (11 cases) as moderate and 8.62% (10 cases) as severe. The average dwell time of the catheter at the time of CASI occurrence was 66.50 days (29.00, 111.50). In the temporal validation cohort of 568 patients, the incidence of PICC‐related CASI was 7.04% (40 cases). Among these cases, 82.50% (33 cases) were mild, 7.50% (three cases) were moderate and 10.00% (four cases) were severe. The average dwell time of the catheter at the time of CASI occurrence was 65.00 days (36.00, 130.00). Baseline characteristics of participants with and without PICC‐related CASI in the development cohort are presented in Table 1.

**TABLE 1:** Baseline characteristics of participants with and without PICC‐related CASI (*n* = 1309).

Independent variable	Classification	No PICC‐related CASI occurred (*n* = 1193)	Occurrence of PICC‐related CASI (*n* = 116)
Age (*n*, %)	< 40 years	80 (6.70)	10 (8.60)
	40–60 years	386 (32.40)	50 (43.10)
	> 60 years	727 (60.90)	56 (48.30)
Gender (*n*, %)	Female	487 (40.80)	54 (46.60)
	Male	706 (59.20)	62 (53.40)
Degree of education (*n*, %)	Illiterate	168 (14.10)	10 (8.60)
	Primary School	395 (33.10)	34 (29.30)
	Junior High School or Vocational School	359 (30.10)	43 (37.10)
	Senior High School	210 (17.60)	23 (19.80)
	Bachelor's Degree and Above	61 (5.10)	6 (5.20)
Height [cm, M (P25, P75)]		165.00 (158.00, 170.00)	163.00 (158.00, 170.00)
Weight [kg, M (P25, P75)]		60.00 (53.00, 69.00)	61.25 (55.09, 68.00)
Smoking history (*n*, %)	No	747 (62.60)	77 (66.40)
	Yes	446 (37.40)	39 (33.60)
Drinking history (*n*, %)	No	743 (62.30)	75 (64.70)
	Yes	450 (37.70)	41 (35.30)
Allergy history (*n*, %)	No	1031 (86.40)	96 (82.80)
	Yes	162 (13.60)	20 (17.20)
Diagnosis (*n*, %)	Respiratory System Tumours	154 (12.90)	27 (23.30)
	Urinary System Tumours	10 (0.80)	1 (0.90)
	Immune System Tumours	8 (0.70)	0 (0.00)
	Endocrine System Tumours	3 (0.30)	0 (0.00)
	Nervous System Tumours	5 (0.40)	1 (0.90)
	Reproductive System Tumours	119 (10.00)	27 (23.30)
	Digestive System Tumours	414 (34.70)	46 (39.70)
	Circulatory System Tumours	53 (4.40)	8 (6.90)
	Locomotor System Tumours	1 (0.10)	1 (0.90)
	Other Non‐Neoplastic Diseases	426 (35.70)	5 (4.30)
Hypertension, (*n*, %)	No	790 (66.20)	80 (69.00)
	Yes	403 (33.80)	36 (31.00)
Diabetes, (*n*, %)	No	994 (83.30)	108 (93.10)
	Yes	199 (16.70)	8 (6.90)
Kidney disease (*n*, %)	No	1134 (95.10)	115 (99.10)
	Yes	59 (4.90)	1 (0.90)
BMI (*n*, %)	18.5–23.9	844 (70.70)	100 (86.2)
	< 18.5	190 (15.90)	10 (8.60)
	24–27.9	61 (5.10)	0 (0.00)
	≥ 28.0	98 (8.20)	6 (5.20)
Serum albumin, (*n*, %)	≥ 34 g/L	724 (60.70)	100 (86.20)
	< 34 g/L	469 (39.30)	16 (13.80)
Haemoglobin (*n*, %)	≥ 130 g/L	309 (25.90)	23 (19.80)
	< 130 g/L	884 (74.10)	93 (80.20)
Catheter insertion season (*n*, %)	Spring and Autumn	630 (52.80)	57 (49.10)
	Summer and Winter	563 (47.20)	59 (50.90)
Space for catheter insertion (*n*, %)	Operating Room	767 (64.30)	108 (93.10)
	Bedside	426 (35.70)	8 (6.90)
Insertion location (*n*, %)	Upper arm	1185 (99.30)	115 (99.10)
	Legs	8 (0.70)	1 (0.90)
Limb selection (*n*, %)	Left	491 (41.20)	57 (49.10)
	Right	702 (58.80)	59 (50.90)
Vein (*n*, %)	Basilic vein	950 (79.60)	97 (83.60)
	Brachial vein	229 (19.20)	18 (15.50)
	Cephalic vein	6 (0.50)	0 (0.00)
	Femoral vein	8 (0.70)	1 (0.90)
Puncture time (*n*, %)	1time	1151 (96.50)	111 (95.70)
	≥ 2time	42 (3.50)	5 (4.30)
Catheter diameter (*n*, %)	4F	657 (55.10)	101 (87.10)
	5F	530 (44.40)	15 (12.90)
	6F	6 (0.50)	0 (0.00)
Catheter material (*n*, %)	Polyurethane	615 (51.60)	33 (28.40)
	Silicone	578 (48.40)	83 (71.60)
Catheter type (*n*, %)	Non‐Power injection	578 (48.40)	83 (71.60)
	Power injection	615 (51.60)	33 (28.40)
History of catheterization (*n*, %)	0 time	1024 (85.80)	88 (75.90)
	≥ 1 time	169 (14.20)	28 (24.10)
History of same limb catheterization (*n*, %)	0 time	1133 (95.00)	105 (90.50)
	≥ 1time	60 (5.00)	11 (9.50)
Functional dressing (*n*, %)	No	1035 (86.80)	108 (93.10)
	Yes	158 (13.2)	8 (6.90)
Iodophor disinfectant (*n*, %)	No	896 (75.10)	109 (94.00)
	Yes	297 (24.90)	7 (6.00)
Chlorhexidine disinfectant (*n*, %)	No	34 (2.80)	2 (1.70)
	Yes	1159 (97.20)	114 (98.30)
Elastic bandage (*n*, %)	No	847 (71.00)	78 (67.20)
	Yes	346 (29.00)	38 (32.80)
Self‐adhesive bandage (*n*, %)	No	1012 (84.80)	95 (81.90)
	Yes	181 (15.20)	21 (18.10)
Cytotoxic drugs (*n*, %)	No	553 (46.40)	9 (7.80)
	Yes	640 (53.60)	107 (92.20)
Molecular targeted drugs (*n*, %)	No	946 (79.30)	74 (63.80)
	Yes	247 (20.70)	42 (36.20)
Immunotherapy drugs (*n*, %)	No	1126 (94.40)	111 (95.70)
	Yes	67 (5.60)	5 (4.30)
Hormonal drugs (*n*, %)	No	441 (37.00)	11 (9.50)
	Yes	752 (63.00)	105 (90.50)
Liposome drugs (*n*, %)	No	1137 (95.30)	104 (89.70)
	Yes	56 (4.70)	12 (10.30)

### 2.3 Variable Selection Based on the LASSO Regression

LASSO logistic regression with 10‐fold cross‐validation was used for predictor selection. The penalty parameter was determined using the one‐standard‐error criterion (lambda.1se), which was selected to obtain a more parsimonious model. At the selected value of lambda, seven variables had non‐zero coefficients: primary diagnosis, history of catheterization, catheter diameter, iodophor disinfectant use, functional dressing use, cytotoxic drug use and hormonal drug use. The cross‐validation curve and coefficient paths are presented in Figure 1.

**FIGURE 1:**
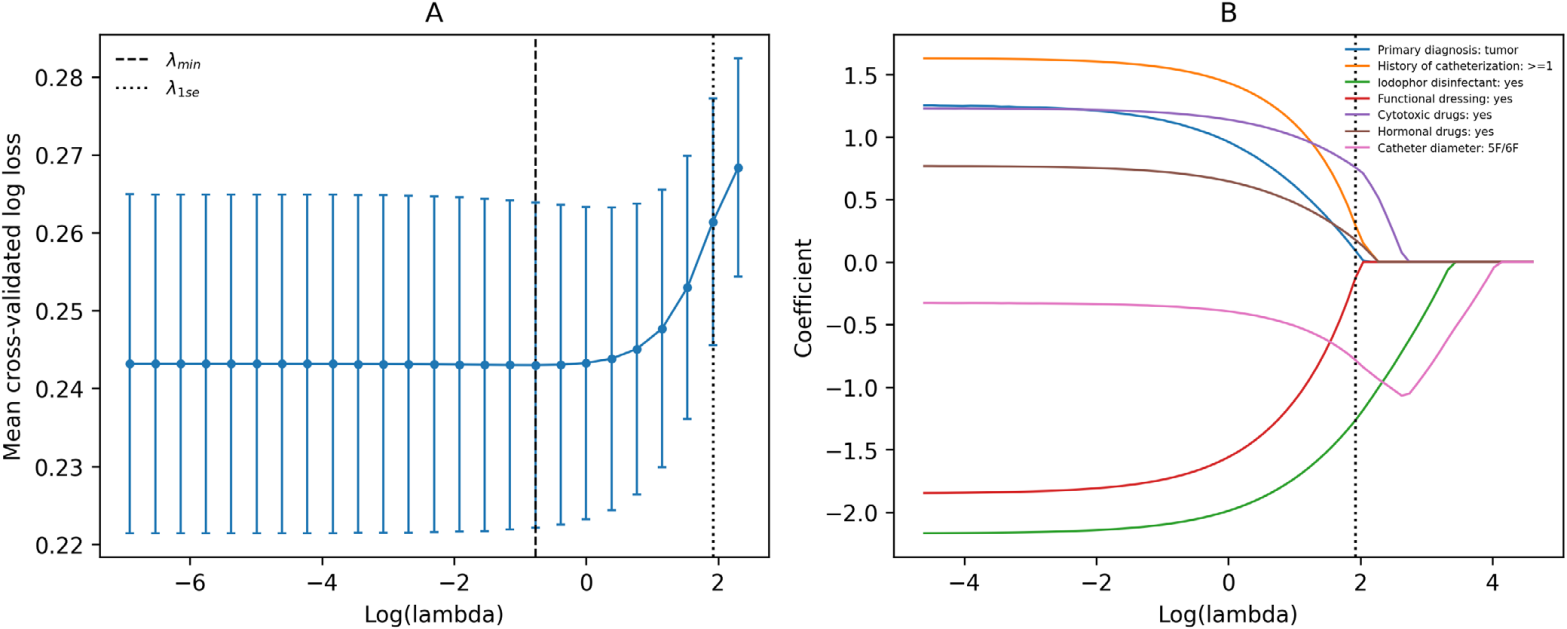
Predictor selection using LASSO logistic regression. (A) Ten‐fold cross‐validation curve for determining the optimal penalty parameter. The dashed line represents lambda.min and the dotted line represents lambda.1se, which was selected for predictor selection. The upper axis indicates the number of non‐zero coefficients. (B) LASSO coefficient profiles of the candidate predictors. Seven predictor variables retained non‐zero coefficients at lambda.1se.

### 2.4 Multifactorial Logistic Regression Analysis Results

The seven LASSO‐selected variables were entered into an unpenalized multivariable logistic regression. Catheter diameter contributed little additional independent information in this refit and the 6F category was very sparse (six participants and no CASI events), producing unstable estimation. It was therefore omitted as a post‐LASSO parsimony decision rather than on the basis of a single significance threshold alone. The final six‐predictor model included primary tumour diagnosis, history of catheterization, iodophor disinfectant use, functional dressing use, cytotoxic drug use and hormonal drug use (Table 2). The fitted equation was as follows: logit(p) = −4.940 + 1.351(tumour) + 1.626(history of catheterization)−2.178(iodophor)−1.822(functional dressing) + 1.394(cytotoxic drugs) + 0.775(hormonal drugs), with binary indicators coded 1 for the stated category and 0 for the reference category.

**TABLE 2:** Final six‐predictor logistic regression model in the development cohort (*n* = 1309).

Predictor	B	SE	OR	95% CI	*p*	Coding (1 vs. 0)
Primary tumour diagnosis	1.351	0.557	3.862	1.297–11.504	0.015	Cancer vs. non‐cancer
History of catheterization	1.626	0.328	5.085	2.673–9.671	< 0.001	≥ 1 vs. 0
Iodophor disinfectant	−2.178	0.404	0.113	0.051–0.250	< 0.001	Yes vs. no
Functional dressing	−1.822	0.478	0.162	0.063–0.413	< 0.001	Yes vs. no
Cytotoxic drugs	1.394	0.440	4.030	1.700–9.552	0.002	Yes vs. no
Hormonal drugs	0.775	0.354	2.172	1.085–4.345	0.028	Yes vs. no

*Note:* Reference categories: non‐tumour diagnosis, no history of catheterization, no iodophor disinfectant, no functional dressing, no cytotoxic drugs and no hormonal drugs.

Abbreviations: CI, confidence interval; OR, odds ratio.

### 2.5 Development of the Model for Predicting the Risk of PICC‐Related CASI

The final six‐predictor logistic model was visualized as a relative predictor score plot (Figure 2). Protective factors are explicitly labelled and coded so that their presence contributes fewer points. For example, a patient with a tumour diagnosis, history of catheterization, cytotoxic and hormonal drug exposure, but without iodophor disinfectant or a functional dressing, receives a substantially higher total score than a patient without these risk factors and with both protective care measures. The corresponding probability should be calculated using the reported logistic equation.

**FIGURE 2:**
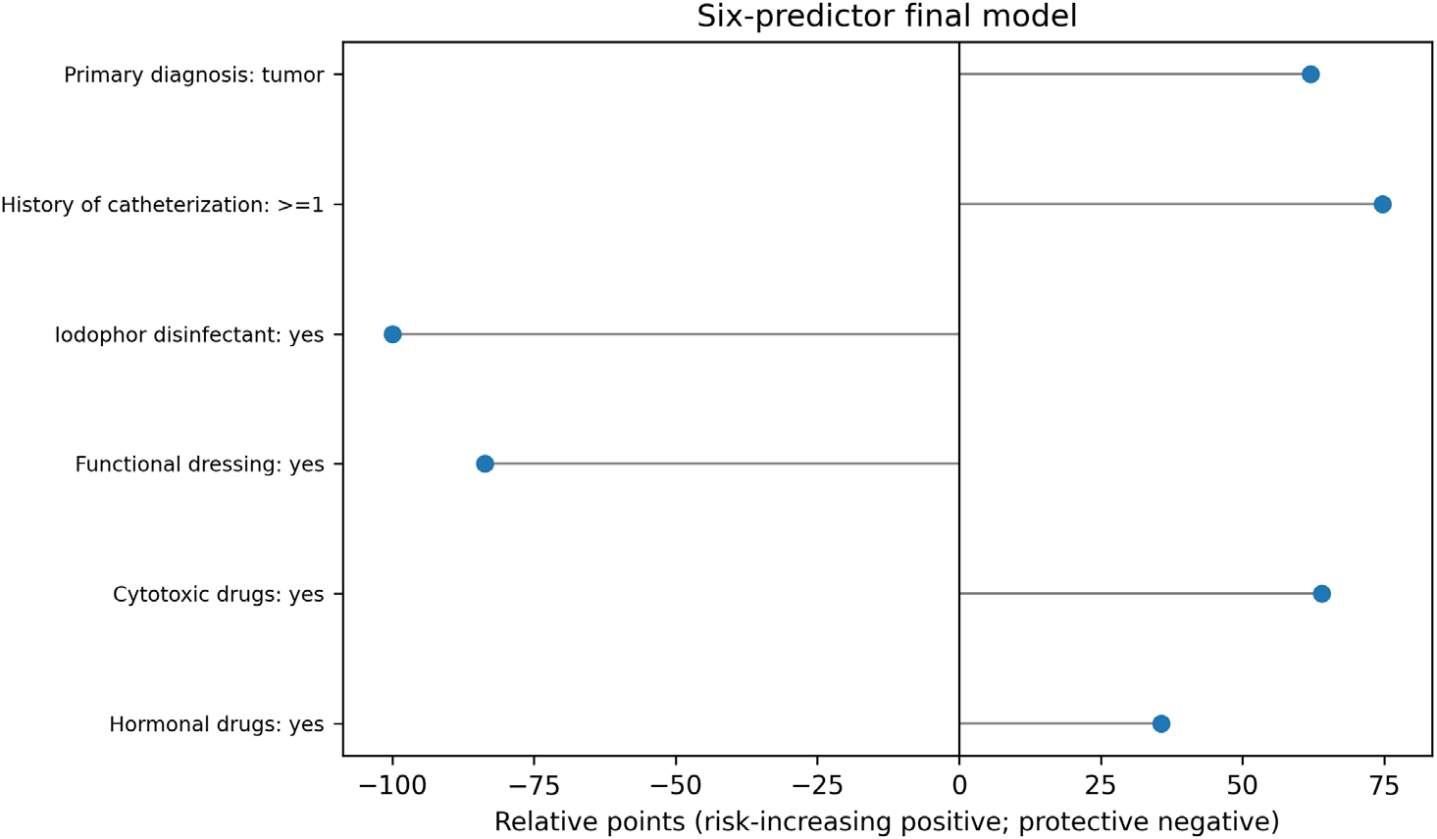
Relative predictor score plot of the final six‐predictor model.

### 2.6 Prediction Model Validation

Bootstrap internal validation with 1000 resamples yielded an optimism‐corrected AUC of 0.811 in the development cohort (95% CI 0.784–0.853) (Figure 3). This should be interpreted as internally corrected development performance rather than as out‐of‐sample validation. The model showed close apparent calibration in the development cohort; the bootstrap‐corrected calibration slope was 0.943. In the temporal validation cohort (*n* = 568; 40 events), the AUC was 0.681 (95% CI 0.610–0.743) (Figure 3), representing a material reduction in discrimination. The calibration intercept was −0.668 and the calibration slope was 0.447 (Figure 4). The calibration curve was close to the ideal line at lower predicted risks but fell below it at higher predicted risks, indicating overprediction and limited separation of higher‐risk patients. The upper‐risk region should be interpreted cautiously because it was supported by relatively few events. Calibration plots, intercepts and slopes were prioritized over the Hosmer‐Lemeshow test.

**FIGURE 3:**
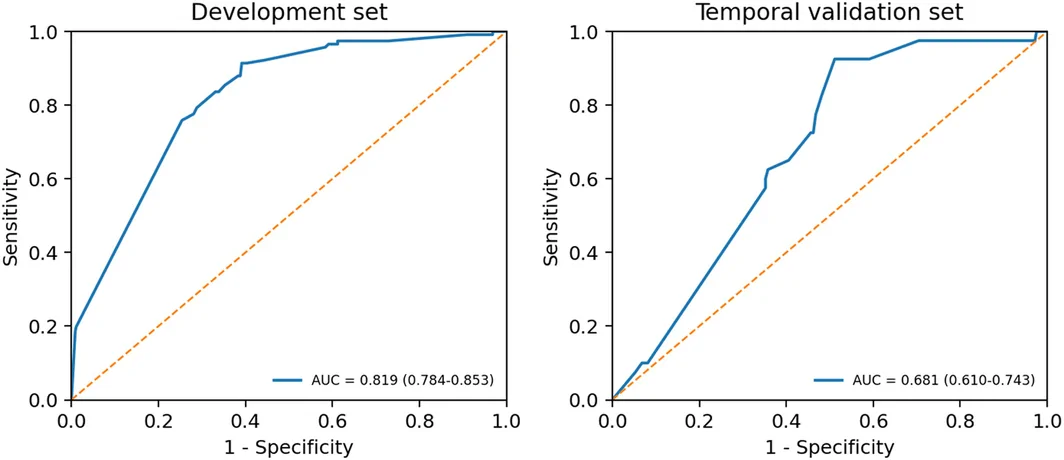
Receiver operating characteristic curves for the development and temporal validation cohorts.

**FIGURE 4:**
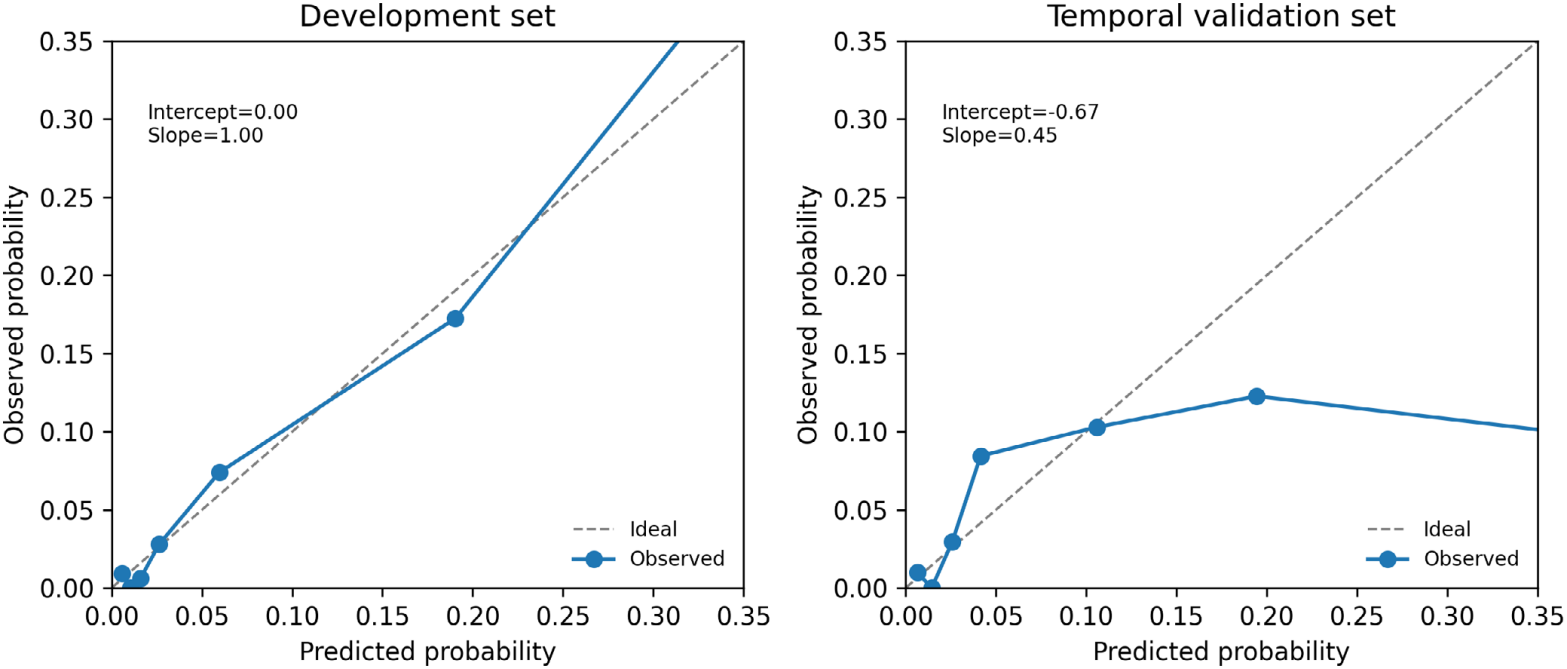
Calibration curves for the development and temporal validation cohorts. The validation curve demonstrates overprediction at higher predicted probabilities; the calibration intercept was −0.668 and the slope was 0.447.

Decision curve analysis was performed in both the development and temporal validation cohorts (Figure 5). Net benefit was compared with treat‐all and treat‐none strategies across clinically plausible threshold probabilities. Because the validation cohort contained only 40 events and utility varied across the threshold range, the curves should be interpreted as exploratory. A threshold for clinical action should be prespecified and tested prospectively rather than selected solely from the development cohort.

**FIGURE 5:**
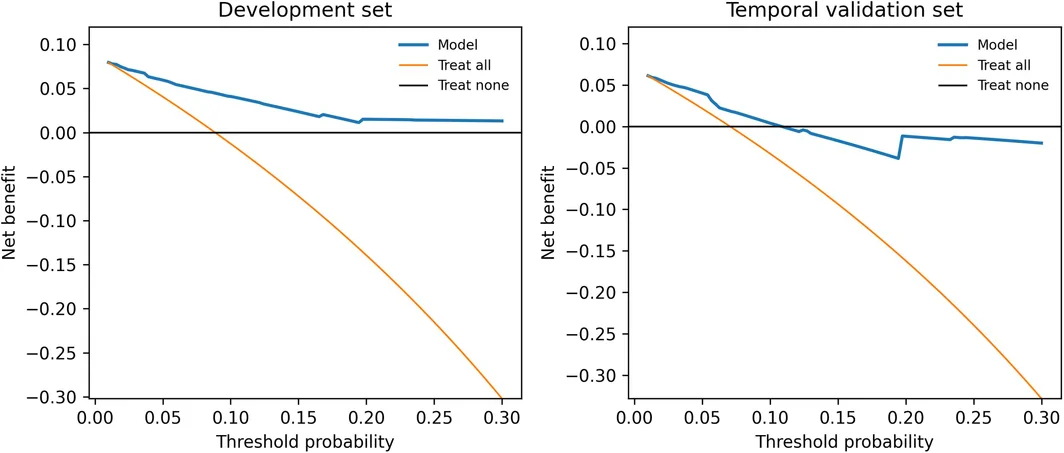
Decision curve analysis in the development and temporal validation cohorts. Net benefit is shown relative to treat‐all and treat‐none strategies across threshold probabilities.

## 3 Discussion

In this prospective single‐centre cohort of 1877 patients, the overall incidence of PICC‐related CASI was 8.31%. The incidence was 8.86% in the development cohort and 7.04% in the temporal validation cohort. These rates were lower than those reported in some previous studies of vascular access device‐associated skin complications (Ullman et al. 2019; Wang et al. 2023). One possible explanation is that earlier studies frequently focused on patients with cancer or other populations with a relatively high baseline risk of skin complications, whereas the present study included both patients with tumours and those with non‐tumour conditions. In addition, all PICCs in this study were inserted and maintained by a specialist intravenous therapy team using relatively standardized procedures. Such local practices may have reduced the occurrence of CASI (Broadhurst et al. 2017; Vilão et al. 2024; Maiwald and Chan 2012).

The final prediction model included six factors: primary tumour diagnosis, history of catheterization, iodophor disinfectant use, functional dressing use, cytotoxic drug use and hormonal drug use. The model therefore incorporated both patient‐ and treatment‐related risk factors and potentially protective catheter‐maintenance factors. This differs from previous models that focused mainly on medical adhesive‐related skin injury or selected groups of patients with cancer (Li, Zhang, et al. 2023; Li, Hao, et al. 2023). However, direct comparison with previous studies should be made cautiously because definitions of skin injury, study populations, clinical settings and predictor measurements were not identical. We acknowledge that refitting an unpenalized logistic regression after LASSO selection may introduce additional selection uncertainty. However, this approach was adopted to improve model interpretability and facilitate clinical implementation. This modelling choice and its limitations should be considered when interpreting the results (Collins et al. 2024; Wolff et al. 2019).

A recent 2025 study developed a CVC‐CASI prediction model in adult ICU patients and identified several patient‐ and catheter‐related predictors, including catheter dwell time, catheterization site, skin edema, serum albumin and puncture frequency (Huang et al. 2025). In comparison, the present study focused specifically on PICCs in a heterogeneous hospitalized population and additionally considered treatment‐related and catheter‐maintenance factors. These differences in device type, patient population and predictor composition highlight the distinct clinical contexts of the two models.

The model showed good apparent discrimination in the development cohort, with an optimism‐corrected AUC of 0.811 after bootstrap validation. However, discrimination decreased to 0.681 in the temporal validation cohort. The validation calibration intercept of −0.668 and calibration slope of 0.447 also suggested systematic overprediction and overfitting. The calibration curve was relatively close to the ideal line at lower predicted probabilities but deviated from it at higher probabilities, where the model tended to overestimate the observed risk. Therefore, the model should be regarded as a development‐stage prediction tool with potential value for local risk stratification and recalibration and independent external validation are required before implementation (Collins et al. 2024; Wolff et al. 2019). Differences in case mix, seasonal recruitment (summer months: July to September), treatment exposure and local antiseptic or dressing practices may have contributed to the decrease in performance; temperature and seasonal variation may influence skin‐barrier function and adhesive‐related irritation (Hui‐Beckman et al. 2023).

History of catheterization was associated with higher odds of PICC‐related CASI in the final model (OR 5.08, 95% CI 2.67–9.67). This finding is generally consistent with previous evidence showing that patients with repeated PICC placement may experience more catheter‐related complications (Cao et al. 2020; Nie et al. 2024). Patients with a history of catheterization may have fewer suitable veins or limbs available for subsequent access and may be exposed to repeated skin disinfection, adhesive application and dressing removal. Repeated adhesive application and removal may increase mechanical irritation of the skin (Tokumura et al. 2005). Prolonged dressing coverage may also create a relatively moist local environment and increase repeated mechanical and chemical stimulation of the skin. In addition, some patients requiring repeated catheterization have received multiple courses of anticancer treatment, which may adversely affect baseline skin integrity. Alternative long‐term vascular‐access strategies may warrant evaluation in selected patients who require repeated access (Tashi et al. 2024).

Iodophor disinfectant use was associated with lower odds of PICC‐related CASI in the present cohort (OR 0.11, 95% CI 0.05–0.25). During PICC insertion and maintenance, antiseptic solutions are repeatedly applied to the skin surrounding the catheter. In this study, commonly used antiseptic regimens included alcohol‐containing chlorhexidine and iodophor‐based products, with some patients receiving iodophor without alcohol because of intolerance. Chlorhexidine‐alcohol is widely recommended for the prevention of catheter‐related infection, but irritant or allergic skin reactions to antiseptic preparations have been reported (Nickel et al. 2024; Lachapelle 2014). Evidence from the CLEAN trial showed more severe skin reactions with chlorhexidine‐alcohol than with povidone‐iodine‐alcohol; however, that trial involved short‐term central venous catheters and focused primarily on infectious outcomes rather than PICC‐related CASI (Mimoz et al. 2015). The contribution of alcohol to the efficacy of combination antiseptic preparations should also be considered when interpreting comparisons between chlorhexidine‐alcohol and iodine‐alcohol products (Maiwald and Chan 2012). Accordingly, the present finding should be interpreted cautiously because the trial population and clinical context differed from those of the present study. These findings suggest that povidone‐iodine may be considered for selected patients at high risk of skin injury, although prospective comparative studies are needed before changes to routine practice are recommended.

Functional dressing use was also associated with lower odds of CASI (OR 0.16, 95% CI 0.06–0.41). The functional dressings used in this study mainly included thin foam and hydrocolloid dressings, whereas conventional transparent film dressings generally consisted of polyurethane film and acrylic adhesive. Foam and hydrocolloid materials may help manage local moisture, reduce friction and shear and provide a protective interface for fragile skin. These properties may explain the lower occurrence of CASI among patients receiving functional dressings. Systematic‐review evidence indicates that dressing and securement choices influence central venous catheter‐associated complications, while hydrogel and related protective materials may support the management of inflammation and skin injury (Xu et al. 2024; Huang et al. 2022). Functional dressings may therefore be considered for patients at high risk of PICC‐related CASI, while recognizing that this observational association requires prospective confirmation.

Cytotoxic drug use was associated with higher odds of PICC‐related CASI (OR 4.03, 95% CI 1.70–9.55). Cytotoxic agents can produce a variety of cutaneous adverse effects, including erythema, papules, oedema, bullae, dryness and impairment of skin‐barrier function (Haynes and Ortega‐Loayza 2020; Wan et al. 2021). Patients receiving these agents may also have reduced immunity, poor nutritional status, gastrointestinal symptoms or general physical weakness, all of which may increase susceptibility to local skin injury. The catheter site is additionally exposed to repeated antisepsis, adhesive dressings and mechanical stress, which may aggravate pre‐existing treatment‐related skin vulnerability. Previous multicentre evidence has also identified chemotherapy exposure as a factor associated with PICC‐related complications, including skin‐related complications (Nie et al. 2024). Clinically, patients receiving cytotoxic therapy may benefit from closer observation of the catheter‐site skin and timely adjustment of dressing or maintenance practices when early signs of irritation are observed.

Hormonal drug use was associated with higher odds of PICC‐related CASI (OR 2.17, 95% CI 1.09–4.35). The hormonal drugs included in this study comprised sex hormones and corticosteroids. Long‐term or high‐dose corticosteroid exposure may contribute to skin thinning, impaired collagen synthesis, delayed repair and greater susceptibility to infection or mechanical injury (Mcdonough et al. 2008). Some hormonal regimens may also be markers of underlying disease severity or treatment intensity. Therefore, the observed association may reflect both direct drug effects and residual confounding related to treatment indication. The duration, cumulative dose, route of administration and type of hormonal drug were not modelled separately in the present analysis. It is therefore not possible to determine whether the association was driven mainly by corticosteroids, sex hormones, treatment duration or underlying disease. Patients receiving prolonged or intensive hormonal treatment may warrant more frequent assessment of local skin integrity.

A primary tumour diagnosis was associated with higher odds of PICC‐related CASI than a non‐tumour diagnosis (OR 3.86, 95% CI 1.30–11.50). Patients with cancer frequently require prolonged vascular access for chemotherapy, supportive treatment, parenteral nutrition or repeated blood sampling (Wang et al. 2023; Li, Hao, et al. 2023; Gavin et al. 2024). Cancer itself, anticancer therapy, radiotherapy, nutritional impairment, immune dysfunction and reduced skin‐barrier function may all contribute to increased vulnerability; anticancer therapies are also associated with a broad spectrum of cutaneous adverse effects (Haynes and Ortega‐Loayza 2020; Wan et al. 2021). The tumour‐diagnosis variable may overlap with cytotoxic and hormonal drug exposure and multicentre studies have reported several treatment‐related risk factors for PICC complications in oncology populations (Nie et al. 2024). Although these factors remained in the final multivariable model, residual confounding and complex relationships among disease type, treatment regimen and baseline skin condition are possible. Further research in oncology populations should examine individual cancer types, treatment protocols, radiation exposure, nutritional status and other supportive therapies to clarify their respective contributions.

The decision‐curve analysis suggested that the model may provide net benefit over treat‐all and treat‐none strategies across parts of the evaluated threshold range in the development cohort. However, the clinical utility was less consistent in the temporal validation cohort, which included only 40 CASI events. Thus, the decision‐curve results should be regarded as exploratory and they should not be interpreted as evidence for immediate clinical implementation (Collins et al. 2024; Wolff et al. 2019). A clinically meaningful threshold for intervention has not yet been established. Future studies should prospectively define plausible risk thresholds and link them to specific preventive actions, such as enhanced skin assessment, alternative antiseptic selection or use of protective dressings.

Overall, the study provides a convenient way to summarize several clinically relevant predictors and estimate individual CASI probability. Its principal potential value lies in supporting structured assessment and raising awareness of modifiable care‐related factors.

### 3.1 Implications for Nursing Practice

Nurses have a central role in the prevention and early recognition of PICC‐related skin complications because they routinely assess catheter sites, perform catheter maintenance and monitor changes in patients' treatment and skin condition. The findings of this study draw attention to several aspects that may be relevant when assessing patients with PICCs, including underlying disease, previous catheterization, anticancer treatment plans and planned catheter‐maintenance practices. Awareness of these factors may support a more structured approach to skin assessment and help nurses identify patients who warrant particularly careful surveillance during PICC care.

However, the prediction model remains at the development stage and should not currently be used to determine individual nursing interventions or product selection. Further recalibration and independent multicentre validation are needed to establish whether the model can reliably support nursing assessment across different clinical settings. Future research should also determine appropriate risk thresholds and evaluate whether incorporating the model into nursing assessment pathways can improve early detection of CASI and patient outcomes.

### 3.2 Limitations of This Study

This study has several important limitations. First, it was conducted at a single tertiary hospital using convenience sampling and all PICCs were managed by a specialist intravenous therapy team. Therefore, local patient characteristics and catheter‐maintenance practices may limit generalizability. Second, no formal model‐development sample‐size calculation was performed before recruitment and the number of CASI events was relatively limited compared with the number of candidate predictor parameters. Although LASSO regression and bootstrap validation were used, some degree of overfitting may remain. The LASSO selection step was not repeated inside the bootstrap loop and the subsequent unpenalized refit and post‐LASSO omission of catheter diameter introduce additional selection uncertainty. Model performance decreased in the temporal validation cohort, with the AUC declining from 0.811 in the development cohort to 0.681 and the calibration slope decreasing to 0.447. These findings indicate that the model may overpredict risk and that its transportability remains uncertain. In addition, categorization of continuous predictors may have reduced predictive information and no interrater agreement statistic was available for routine CASI classification. Finally, several potentially relevant predictors, including detailed insertion procedures, dressing‐change frequency, product type, baseline skin condition and cumulative medication exposure, were not included. Consistent with current prediction‐model reporting and risk‐of‐bias guidance, independent multicentre validation, recalibration and prospective evaluation of clinical thresholds are required before routine clinical use (Collins et al. 2024; Wolff et al. 2019). The PROBAST risk‐of‐bias and applicability assessment is provided in Table S3.

## Author Contributions

**Xianghong Jin:** methodology, investigation, writing – original draft. **Xiuzhu Cao:** software, conceptualization, methodology, data curation, investigation, writing – original draft, writing – review and editing, validation, formal analysis. **Yidan Chen:** data curation, investigation, validation. **Linfang Zhao:** writing – review and editing, resources, project administration, supervision.

## Funding

This study was supported by the Nursing Research Fund of Sir Run Run Shaw Hospital, Zhejiang University School of Medicine (#2024HLKY06) and the Zhejiang Provincial Medical and Health Science and Technology Plan (General Program) (#2022KY178 and #2025KY889).

## Ethics Statement

Ethical approval was obtained from the Ethics Committee of Sir Run Run Shaw Hospital, Zhejiang University School of Medicine, approval No. [2022] (0307). Before the study began, all participants were informed about the purpose and procedures of the study, participation was voluntary and written informed consent was obtained. The study was conducted in accordance with the Declaration of Helsinki.

## Conflicts of Interest

The authors declare no conflicts of interest.

## Supporting information


**Table S1:** Complete list of the 38 prespecified candidate predictors.

**Table S2:** Completed TRIPOD + AI Checklist.
**Table S3:** PROBAST Risk of Bias and Applicability Assessment.

## Data Availability

The datasets generated and/or analysed during the current study are not publicly available because they contain potentially identifiable participant‐level clinical information and are subject to institutional ethical and data‐governance restrictions. Reasonable requests for access to anonymized data may be considered by the corresponding author, subject to approval from the relevant ethics committee and institutional requirements.
