# Supplementary material for: Development and Temporal Validation of a Prediction Model for PICC‐Related Catheter‐Associated Skin Injury
Source: Nurs Open. 2026 Oct 9;13(10):e70895. doi: 10.1002/nop2.70895 (PMC13652349; doi:10.1002/nop2.70895)
Supplement: Supplementary file 1 [file NOP2-13-e70895-s001.docx]

## Supplementary Table S2. Completed TRIPOD+AI Checklist

| Section | Item | Recommendation | Reported | Location |
| --- | --- | --- | --- | --- |
| Title | 1 | Developing and validating prediction model | Yes | Title |
| Abstract | 2 | Objectives, methods, results, validation | Yes | p.1 |
| Background | 3a-3b | Clinical rationale and objectives | Yes | pp.2-3 |
| Source of data | 4a-4b | Design and dates | Yes | pp.3-4 |
| Participants | 5a-5b | Setting and eligibility | Yes | pp.3-4 |
| Outcome | 6a-6b | Outcome definition and assessment | Yes | p.6 |
| Predictors | 7a-7b | Predictor definition and assessment | Yes | pp.4-6 |
| Sample size | 8 | Sample size | Yes | p.4 |
| Missing data | 9 | Missing data | Yes | p.6 |
| Statistical analysis | 10a-10d | Model development and validation | Yes | pp.6, 10-15 |
| Participant flow | 13a | Flow diagram | Yes | p.7; Fig S1 |
| Participant characteristics | 13b | Baseline characteristics | Yes | pp.7-10 |
| Model specification | 15a-15b | Final model and equation | Yes | pp.11-12 |
| Model performance | 16 | AUC, calibration, DCA | Yes | pp.13-15 |
| Discussion | 18-20 | Limitations and interpretation | Yes | pp.15-21 |

## Supplementary Table S3. PROBAST Risk of Bias and Applicability Assessment

| Domain | Signalling question | Judgment | Location | Comments |
| --- | --- | --- | --- | --- |
| Participants | Appropriate participants? | Low | pp.3-4 | Prospective cohort |
| Predictors | Clearly defined? | Low | pp.4-6 | Prespecified predictors |
| Outcome | Clearly defined? | Low | p.6 | INS + Broadhurst |
| Outcome | Assessment appropriate? | Some concerns | p.6 | No kappa statistic |
| Analysis | Sample size adequate? | Some concerns | p.4 | No pmsampsize |
| Analysis | Missing data handled? | Low | p.6 | Complete-case |
| Analysis | Model development appropriate? | Low | pp.10-12 | LASSO + logistic |
| Analysis | Validation appropriate? | Low | pp.13-15 | Bootstrap + temporal validation |
| Overall | Risk of bias | Moderate | - | Limited events/possible overfitting |
| Applicability | Applicability | Low-Moderate concern | - | Single-centre study |
