# Supplementary material for: Development and Temporal Validation of a Prediction Model for PICC‐Related Catheter‐Associated Skin Injury
Source: Nurs Open. 2026 Oct 9;13(10):e70895. doi: 10.1002/nop2.70895 (PMC13652349; doi:10.1002/nop2.70895)
Supplement: Supplementary file 2 [file NOP2-13-e70895-s002.docx]

**Supplementary Table S1. Complete list of the 38 prespecified candidate predictors**

Intended prediction time: PICC insertion after formulation of the initial maintenance plan. Prediction horizon: from PICC insertion to the first PICC-related CASI, PICC removal, or 6 months, whichever occurred first.

| **No.** | **Candidate predictor** | **Operationalization in analysis dataset** | **Assessment/source relative to prediction time** | **Available at intended prediction time?** |
| --- | --- | --- | --- | --- |
| 1 | Sex | Female / male | Baseline demographic record | Yes |
| 2 | Height | Continuous, cm | Baseline/first study visit | Yes |
| 3 | Weight | Continuous, kg | Baseline/first study visit | Yes |
| 4 | Education | Illiterate; primary; junior/vocational; senior high; bachelor or above | Baseline demographic record | Yes |
| 5 | Smoking history | No / yes | Baseline history | Yes |
| 6 | Alcohol use history | No / yes | Baseline history | Yes |
| 7 | Allergy history | No / yes | Baseline history | Yes |
| 8 | Hypertension | No / yes | Baseline medical history | Yes |
| 9 | Diabetes | No / yes | Baseline medical history | Yes |
| 10 | Kidney disease | No / yes | Baseline medical history | Yes |
| 11 | Molecular targeted therapy | No / yes | Ongoing treatment or established treatment plan documented at PICC insertion; subsequent administration used only for confirmation | Yes |
| 12 | Immunotherapy | No / yes | Ongoing treatment or established treatment plan documented at PICC insertion; subsequent administration used only for confirmation | Yes |
| 13 | Liposome drug use | No / yes | Ongoing treatment or established treatment plan documented at PICC insertion; subsequent administration used only for confirmation | Yes |
| 14 | Nutritional intake status | Normal; mild; moderate; severe impairment | Clinical assessment around insertion | Yes, if based on insertion assessment |
| 15 | Catheter insertion setting | Catheterization room / ward (bedside) | At PICC insertion | Yes |
| 16 | History of catheterization | 0 vs ≥1 prior catheterization | Baseline history | Yes |
| 17 | History of same-limb catheterization | 0 vs ≥1 prior same-limb catheterization | Baseline history | Yes |
| 18 | Insertion site | Upper arm / lower limb | At PICC insertion | Yes |
| 19 | Catheterized limb | Left / right | At PICC insertion | Yes |
| 20 | Catheterized vein | Basilic / brachial / cephalic / femoral | At PICC insertion | Yes |
| 21 | Puncture attempts | 1 vs ≥2 | At PICC insertion | Yes |
| 22 | Catheter material | Polyurethane / silicone | At PICC insertion | Yes |
| 23 | Catheter diameter | 4F / 5F / 6F | At PICC insertion | Yes |
| 24 | Smith & Nephew dressing use | No / yes | Initial dressing/maintenance strategy | Yes |
| 25 | 3M dressing use | No / yes | Initial dressing/maintenance strategy | Yes |
| 26 | Iodophor disinfectant use | No / yes | Initial antiseptic/maintenance strategy; post-CASI changes did not redefine baseline status | Yes |
| 27 | Chlorhexidine disinfectant use | No / yes | Initial antiseptic/maintenance strategy | Yes |
| 28 | Elastic bandage use | No / yes | Initial fixation/maintenance strategy | Yes |
| 29 | Self-adhesive bandage use | No / yes | Initial fixation/maintenance strategy | Yes |
| 30 | Catheter insertion season | Spring/autumn vs summer/winter | At PICC insertion | Yes |
| 31 | Age category | <40; 40-59; ≥60 years | Baseline/first study visit | Yes |
| 32 | BMI category | Dataset categories: <18.5; 18.5–23.9; 24.0–27.9; ≥28.0 kg/m² | Baseline BMI calculated from height and body weight available at the first study visit / PICC insertion | Yes |
| 33 | Serum albumin category | <34 vs ≥34 g/L | Baseline serum albumin value available at the first study visit / PICC insertion | Yes |
| 34 | Haemoglobin category | <130 vs ≥130 g/L | Baseline haemoglobin value available at the first study visit / PICC insertion | Yes |
| 35 | Functional dressing use | No / yes | Initial dressing/maintenance strategy; post-CASI changes did not redefine baseline status | Yes |
| 36 | Hormonal therapy | No / yes | Ongoing or established treatment plan at PICC insertion; administration prospectively confirmed | Yes |
| 37 | Cytotoxic therapy | No / yes | Ongoing or established treatment plan at PICC insertion; administration prospectively confirmed | Yes |
| 38 | Primary diagnosis | Tumor / non-tumor | Baseline diagnosis | Yes |
